# Computational MHC-I epitope predictor identifies 95% of experimentally mapped HIV-1 clade A and D epitopes in a Ugandan cohort

**DOI:** 10.1186/s12879-020-4876-4

**Published:** 2020-02-22

**Authors:** Daniel Lule Bugembe, Andrew Obuku Ekii, Nicaise Ndembi, Jennifer Serwanga, Pontiano Kaleebu, Pietro Pala

**Affiliations:** 1 MRC/UVRI and LSHTM Uganda Research Unit, P. O. Box 49, Plot 51-59 Nakiwogo Road, Entebbe, Uganda; 2 Institute of Human Virology, Abuja, Nigeria; 3 Uganda Virus Research Institute, Entebbe, Uganda

**Keywords:** HIV-1, Epitope mapping, T-cell, Artificial neural network, *In-silico*, NetMHCpan4.0., MHCflurry1.2.0 and NetCTL1.2

## Abstract

**Background:**

Identifying immunogens that induce HIV-1-specific immune responses is a lengthy process that can benefit from computational methods, which predict T-cell epitopes for various HLA types.

**Methods:**

We tested the performance of the NetMHCpan4.0 computational neural network in re-identifying 93 T-cell epitopes that had been previously independently mapped using the whole proteome IFN-γ ELISPOT assays in 6 HLA class I typed Ugandan individuals infected with HIV-1 subtypes A1 and D. To provide a benchmark we compared the predictions for NetMHCpan4.0 to MHCflurry1.2.0 and NetCTL1.2.

**Results:**

NetMHCpan4.0 performed best correctly predicting 88 of the 93 experimentally mapped epitopes for a set length of 9-mer and matched HLA class I alleles. Receiver Operator Characteristic (ROC) analysis gave an area under the curve (AUC) of 0.928. Setting NetMHCpan4.0 to predict 11-14mer length did not improve the prediction (37–79 of 93 peptides) with an inverse correlation between the number of predictions and length set. Late time point peptides were significantly stronger binders than early peptides (Wilcoxon signed rank test: *p* = 0.0000005). MHCflurry1.2.0 similarly predicted all but 2 of the peptides that NetMHCpan4.0 predicted and NetCTL1.2 predicted only 14 of the 93 experimental peptides.

**Conclusion:**

NetMHCpan4.0 class I epitope predictions covered 95% of the epitope responses identified in six HIV-1 infected individuals, and would have reduced the number of experimental confirmatory tests by > 80%. Algorithmic epitope prediction in conjunction with HLA allele frequency information can cost-effectively assist immunogen design through minimizing the experimental effort.

## Background

Computational algorithms are increasingly utilised in biological modelling and offer the potential to reduce the time and expense of immunological assays. Computational algorithms were initially demonstrated as useful tools for predicting potential epitopes that might elicit quality T-cell responses [1, 2]. Computational algorithms that predict potential HLA binding T-cell epitopes can facilitate the design of vaccines capable of inducing T-cell immunity against HIV-1. The high variability of HIV-1 and the extensive genetic polymorphism of HLA molecules can be managed in silico, allowing immunogen optimisation to increase breadth and magnitude of T cell responses in respect of HLA allele frequencies and circulating virus strains in different populations. Bioinformatics approaches were previously applied as proof of concept for an HIV-1 peptide-based vaccine for the *env* and *gag* genes [3] in cynomolgus macaques for a broad spectrum of HIV-1 clades. Computational optimisation of immunogens facilitates the development of the multivalent and mosaic vaccines [4] necessary to control recombinant HIV-1 strains, an increasingly common occurrence in the epidemic in Uganda [5]. Computational approaches aim to identify optimal epitopes relevant to vaccine development and are not isolated to HIV-1 only, but a wide range of pathogens, including Ebola virus [6], therefore various statistical validation approaches have been applied for evaluation of these methods [7–10].

For HIV-1 vaccine design purposes an important consideration for the suitability of a computational algorithm is the breadth of discrete number of T-cell epitopes it generates that could reach particular levels of coverage [11] of circulating viruses. The higher the number of epitope variants the more the reduction in their requirements to attain optimum coverage levels for any epidemic. Previous data has shown that breadth of T-cell response is associated to viral set point in chronic HIV-1 infection [12–17]. In order to translate the computational epitope prediction into vaccine design, the number of discrete epitopes computationally generated from particular HIV-1 proteins is an important metric for further investigation [11].

A reliable pan-HLA-specific algorithm NetMHCpan4.0 [18–20] that has been improved by advances in HLA binding data, covers 172 MHC class I molecules from human (HLA-A, B, C, E), mouse (H-2), cattle (BoLA), primates (Patr, Mamu, Gogo) and swine (SLA) [20, 21], and can also predict binding to alleles devoid of experimental data basing on similarity to known binders and non-binders [22, 23]. This is an artificial neural network (ANN) algorithm for predictions of 8-14aa and capable of predicting epitopes for other HLA alleles using data for similar alleles by positional similarity of residues in their binding motifs. NetMHCpan4.0 is considered to be the tool of choice for such predictions considering the benchmarking done against other related tools [24]. Nevertheless to have a conclusive outcome of the computational performance we compared NetMHCpan4.0 to both an older and recent tool, NetCTL1.2 [25–27] and MHCflurry1.2.0 [28] respectively. The binding of CTL epitopes to MHC class I molecules is linear, anchoring at residues 2 and 9; hence the interface between ligand and CTL can be determined computationally [29]. Validation of such computational applications can be done by comparing their predictions with suitable experimental data. Despite the paucity of data validating the performance of computational methods relative to wet laboratory experiments, a few have documented them to achieve an area under the curve AUC of over 90% [18, 19, 30, 31] by isolated experimental data. We have not come across a wet experiment that evaluated computational predictors to achieve a robust AUC using a single set of wet laboratory experimental data. The previously reported 90% AUC is largely based on positional specific scoring algorithms (PSSM) for the collective isolated experiments alongside probability models used to establish affinity or binding scores. One study that explored the reliability of *in-silico* approaches in epitope prediction and its application for vaccine design reported a meagre 22, 44%, and relatively higher 78% match for three computational tools namely YFPEITHI, CTLPRED and IEDB respectively [32]. Using experimental epitope mapping data generated from 757 peptides tested on cells of 6 early HIV-1 infected individuals at paired time points, we show that NetMHCpan4.0 can be useful for markedly reducing pooled peptide experiments as demonstrated by the 95% experimental and computational concordance.

## Methods

### Experimental binder data

The data used was from an independent study that did not include this analysis in its objectives. Experimental data of peptides previously mapped for HIV-1 epitope recognition of 6 individuals for a separate study (Table 1) at 2 time points each was used for comparison with the computationally predicted binders. These were from a Ugandan early HIV-1 serodiscordant couple cohort approved by the Uganda Virus Research Institute (UVRI), Research and Ethics review board and the Uganda National Council of Science and Technology (UNCST). All participants provided informed consent. Six (6) participants whose experimental epitope recognition profile we evaluated were early HIV-1 infections (Table 1), enrolled under the following criteria: (i) detection of HIV-1 P24 antigen with a simultaneous negative HIV-1 antibody ELISA (2 participants) or documented HIV-1 sero-negative test in the previous 12 months (4 participants); (ii) HAART naïve (all). Early infection was determined following the Fiebig Staging criteria [33] as described elsewhere by Obuku A.E. *et.al* [34]..

**Table 1:** Participant characteristics, HIV-1 infecting clade, Fiebig stage and HLA class I haplotypes

Subject	Sex	Age range (years)	HIV-1 subtype	Class-I HLA	Early Time Point (Days)	Fiebig Staging	Late Time Point (Days)
91	M	31–40	A	A*0201,*0301;B*5301,*5802;Cw*0401,*0602	121	VI	841
92	F	21–30	D	A*0201,*3002;B*4403,*1402Cw*0401,*0802	52	VI	743
94	M	51–60	A	A*3402,*7401;B*4403,*5802;Cw*0401,*0602	28	V	358
95	M	21–30	A	A*2301,*7401;B*4403,*1510;Cw*0401,*1601	30	VI	570
913	F	11–20	D	A*0201,*3402;B*4501,*4701;Cw*0602,*1601	61	VI	211
914	F	21–30	D	A*0101,*0201;B*0702,*4415;Cw*0407,*0702	31	IV	181

**Fig. 1:**
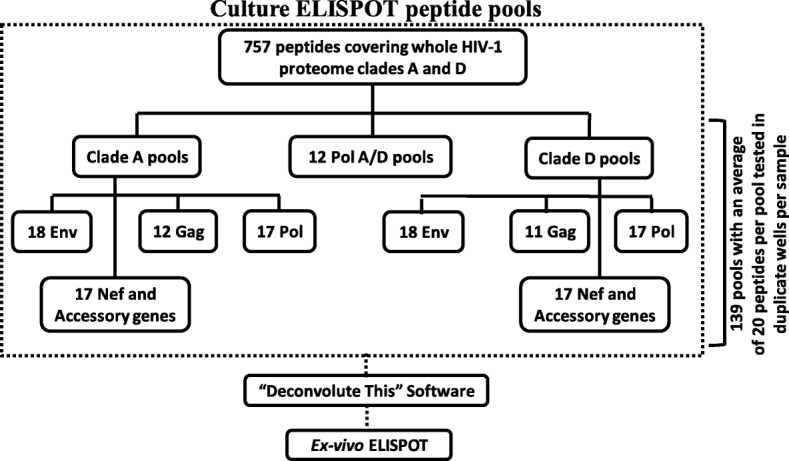
ELISPOT peptide consort; the experimental peptide mapping data was generated by culture ELISPOT of multiple peptide pools tested in duplicate wells per time point, followed by ex-vivo ELISPOT of potential candidate epitopes. To experimentally map a single time point required at least 541 assay wells

The experimentally tested peptides totalled 757 (Fig. 1), were 17aa long, overlapping by 11aa and spanning the HIV-1 proteome consensus for subtypes A1 and D. Cultured ELISPOT assays using 200,000 cells/well as previously documented by Obuku AE. *et.al* [34]. and ex-vivo IFN-γ ELISPOT assay using 100,000 cells/well were used for testing peptide pools and epitope mapping respectively. Experimental positive pools were 3 times the background wells and at least 600 spot forming units per million cells. “Deconvolute This” software [35] was used to identify possible responding individual peptides from the pools or where it was not possible all the peptides in a pool were tested as single peptides.

### HLA typing

High resolution reference strand conformation analysis HLA class I tissue typing for the early infected subjects was done using methods described elsewhere [36].

### HIV-1 subtyping

HIV-1 subtyping determination was performed on the *gag* gene [37, 38] using Sanger method generated sequences. The sequences were input into the REGA HIV-1 automated subtyping tool to determine the HIV-1 clade [39, 40].

### Computation epitope prediction

HIV-1 subtypes A1 and D consensus sequences were used as inputs for the computational epitope prediction. These peptide sequences were all for the year 2004 downloaded from the Los Alamos database (hiv.lanl.gov/content/sequence/NEWALIGN/align.html). The web version of NetMHCpan4.0 [19] (http://www.cbs.dtu.dk/services/NetMHCpan/) was configured to predict 9mer through 14mer epitopes for 22 HLA class I alleles (Table 1) that were expressed by the 6 HIV-1 infected donors. Linux version MHCflurry1.2.0 [28] was used to predict 9mer epitopes and an earlier tool NetCTL1.2 was also used to predict 9mer epitopes for the 22 HLA class I alleles expressed by the 6 study individuals. Perl version 5.26.2 was used to extract the binders from all the NetMHCpan4.0 predictions and also to compare the computational binders to the 93 mapped experimental 17aa peptides for 9mer through 14mer hits using a sliding window. An experimental peptide was considered a hit if any of the computational 9mer through 14mer sequence was contained in the 17 amino acid experimental peptide sequence as well as any of the HLA-A, B or C expressed by the individual matched the NetMHCpan4.0 HLA class I type(s). If multiple computational epitope predictions were contained in a single 17mer experimental peptide they were counted as a single hit. These were determined by a BLAST search of the computational binders against the derivative experimental peptides to determine computational predictions from the same test peptide. The accession numbers of the sequences used to determine the HIV-1 subtypes for 5 of the 6 study subjects are; KT825896, KT825897, KT825898, KT825899, KT825900, KT825901, KT825902, KT825903, KT825904, KT825905, KT825906, KT825907, KT825908, KT825909, KT825910, KT825911 and KT82512.

### Data analysis

Statistics computations and plots were generated using SPSS version 24.0.0.0. The NetMHCpan4.0 computational performance was evaluated using a confusion matrix to classify true positives, true negatives, false positives and false negatives that were used for the Receiver Operator Characteristic (ROC) plot. The hit rate (sensitivity) and false hit rate (specificity) of binder predictions as determined by the NetMHCpan4.0 threshold of peptides within the top 2% (with a score of 2 or less) were calculated and the strength of the model was determined by calculating the area under the curve, AUC of the ROC plot [41–43]. Pearson’s correlation coefficient was used to evaluate the relationship between the number of epitopes with various HIV-1 genes. To evaluate if there were any differences in the early versus late time point peptides for the binding ranking of the experimentally mapped peptides as predicted by the computational score the Wilcoxon signed rank test was used. To evaluate if HIV-1 subtypes A1 and D affected the number of computational predictions generated, Fisher Exact Test was used. To determine whether multiple computationally predicted epitope sequences were derived from the same experimental peptide sequence, a local blast database was set up using Geneious version 9.0.5. Both HIV-1 clades A1 and D experimental consensus sequences were used separately each as a reference sequence for the blast. The computational peptide sequences were then aligned against the consensuses to evaluate those derived from a single 17 amino acid experimental peptide sequence. Where an experimental peptide was predicted by multiple or overlapping computational peptides, the average NetMHCpan4.0 score was assigned as the computational score for this peptide. This score was also used during the generation of the ROC curve and the confusion matrix. To compare the association between ELISPOT spot forming units and NetMHCpan4.0 scores or MHCflurry1.2.0 affinities and also the association between the values for the 2 computational tools, Pearson’s correlation coefficient was used.

## Results

### Number of experimental assays compared to computationally guided prediction assay projections

To experimentally determine epitopes for 757 peptides spanning the whole HIV-1 proteome for clades A and D as well as both time points of the 6 individuals required a total of 4230 test assay wells. For each test subject these included 9 antigen proliferation wells, 384 culture ELISPOT wells and an average of 164 epitope mapping ELISPOT wells (Range; 148–186 test wells). Using the 22 HLA alleles represented in the study subjects we were able to computationally predict 95% of the experimentally mapped epitopes. This approach could have reduced the test assays by eliminating all the T-cell antigen proliferation and culture ELISPOT steps totalling to 3258 assay wells (77%) and leaving only 972 (23%) epitope mapping assays required. Applying a pooling strategy to the computational predictions similar to that used in the experimental pooling where each pool contained approximately 20 peptides with a coverage of 3 per peptide pool, the 923 potential peptides (95% of experimental peptides for epitope mapping ELISPOT derived from the 972 (23%) eligible epitope mapping peptides) would make at most 46 pools. Consequently the computational prediction approach could have reduced the experimental assays by at least 80%.

### Magnitude of epitope predictions are variable across HLA alleles, HIV-1 proteins and clades

**Fig. 2:**
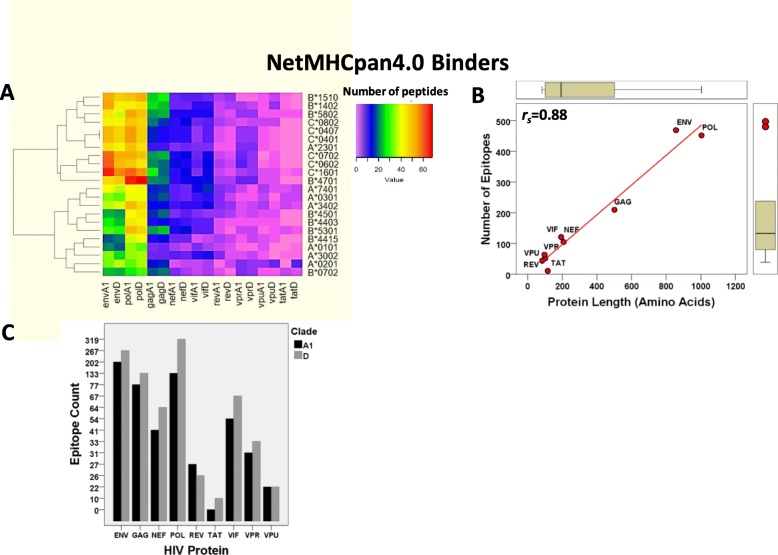
NetMHCpan Binder Predictions. **a** Using our experimental peptide sequences as inputs into NetMHCpan4.0 to predict epitopes for 22 HLA types represented in the 6 HIV-1 Infected people, a heatmap showing absolute counts of computationally predicted 9-mer binders against HIV-1 genes was constructed. The dendrogram shows the nearest similarity for the number of predicted counts across HLA types; **b** the length of the HIV-1 protein sequence plotted against the absolute number of NetMHCpan4.0 predicted 9mer binders showing a positive correlation (Spearman’s correlation coefficient, r_s_ = 0.88). The number of distinct predictions is dependent on the length of the HIV-1 sequence; **c** comparison of HIV-1 clade A and D absolute number of NetMHCpan4.0 predicted 9mer binders per HIV-1 gene for the wet experiment test peptide sequences. The algorithm predicted more binders for clade D than clade A

The input HIV-1 subtypes A1 and D consensus whole proteome sequences evaluated for potential 9, 10, 11, 12, 13 and 14-mer binders to the 22 HLA alleles represented in the six patients, varied in the distribution of predicted binders across HIV-1 genes and HLA alleles. All the peptide hits predicted for 10 through 14-mer were also all predicted in the 9-mer set except for two 14-mer peptides. An expected positive correlation for HIV-1 protein length with number of epitopes predicted was observed as illustrated by Spearman’s rank order correlation; *r*_*s*_ = 0.88 (Fig. 2, a and b). NetMHCpan4.0 predicted 95% (88/93) (Table 2) of the experimentally mapped peptides as binders and missed 5% (5 out of 93) (Table 3) for the 12-time points of the 6 participants. MHCflurry predicted 91% (85/93) of the experimental peptides and had a lot of similarity to NetMHCpan4.0 for the predicted HLA. NetCTL was the least performing tool with only 15% (14/93) predicted experimental peptides (Table 2).

**Table 2:** Experimentally Mapped Peptides and Computationally Predicted Epitopes

ID	Participant’s HLA Types	Hit No	Screening Peptide	Screening Peptide HIV-1 Clade	NetMHCpan4.0 9-mer Epitope Prediction	NetMHCpan4.0 9-mer HLA Prediction	NetMHCpan4.0% Rank	MHCflurry 9-mer Epitope prediction	MHCflurry 9-mer HLA prediction	MHCflurry affinity (μm)	NetCTL 9-mer Epitope prediction
E91	A*02:01	1	FITKGLGISYGRKKRRQR	D	GLGISYGRK	A*0301	0.50	GLGISYGRK	A*0201	0.47	
	A*03:01	2	HPKVSSEVHIPLGDARLV	A1	IPLGDARLV	B*53:01	0.70	IPLGDARLV	A*0302	24.63	
	B*53:01		HPKVSSEVHIPLGDARLV	A1	KVSSEVHIP	B*58:02	0.60	KVSSEVHIP	B*53:01	26.18	
	B*58:02		HPKVSSEVHIPLGDARLV	A1	SSEVHIPLG	B*58:02	0.60				
	Cw*04:01		HPKVSSEVHIPLGDARLV	A1	VSSEVHIPL	Cw*04:01	0.90	VSSEVHIPL	Cw*04:01	18.48	
	Cw*06:02		HPKVSSEVHIPLGDARLV	A1	HPKVSSEVH	B*53:01	1.20	HPKVSSEVH	Cw*06:02	28.58	
E92	A*02:01	3	RKQNPEIVIYQYMDDLYV	D	YQYMDDLYV	A*02:01	0.15	YQYMDDLYV	A*02:01	0.25	
	A*30:02		RKQNPEIVIYQYMDDLYV	D	NPEIVIYQY	B*44:03	0.60	NPEIVIYQY	A*30:02	4.94	
	B*44:03		RKQNPEIVIYQYMDDLYV	D	YQYMDDLYV	A*02:01	1.80	YQYMDDLYV	B*44:03	8.44	
	B*14:02		RKQNPEIVIYQYMDDLYV	D	YQYMDDLYV	Cw*04:01	0.90	YQYMDDLYV	Cw*04:01	1.52	
	Cw*04:01		RKQNPEIVIYQYMDDLYV	D	YQYMDDLYV	Cw*08:02	1.20	YQYMDDLYV	Cw*08:02	13.26	
	Cw*08:02		RKQNPEIVIYQYMDDLYV	D	VIYQYMDDL	A*30:02	0.60	VIYQYMDDL	A*30:02	2.52	
		4	ELNKRTQDFWEVQLGIPH	A1	TQDFWEVQL	Cw*08:02	0.40	YQYMDDLYV	Cw*08:02	3.09	
			ELNKRTQDFWEVQLGIPH	A1	TQDFWEVQL	A*02:01	0.03	TQDFWEVQL	A*02:01	19.72	
			ELNKRTQDFWEVQLGIPH	A1	TQDFWEVQL	Cw*08:02	1.50	TQDFWEVQL	Cw*08:02	3.09	
			ELNKRTQDFWEVQLGIPH	A1	ELNKRTQDF	B*44:03	0.60	ELNKRTQDF	B*44:03	26.56	
		5	NDIQKLVGKLNWASQIYP	D	KLNWASQIY	A*30:02	0.50	KLNWASQIY	A*30:02	0.02	
			NDIQKLVGKLNWASQIYP	D	KLVGKLNWA	A*02:01	0.47	KLVGKLNWA	A*02:01	0.32	
			NDIQKLVGKLNWASQIYP	D	KLNWASQIY	Cw*04:01	1.80	KLNWASQIY	Cw*04:01	2.34	
		6	PAIQTGSEELRSLYNTVA	D	GSEELRSLY	A*30:02	0.40	GSEELRSLY	A*30:02	0.12	
			PAIQTGSEELRSLYNTVA	D	SEELRSLY	B*44:03	0.50	SEELRSLY	B*44:03	16.25	
		7	PYNTPIFAIKKKDSTKWR	A1	PYNTPIFAI	Cw*04:01	1.14	PYNTPIFAI	Cw*04:01	32.80	
		8	GANNSHNETFRPGGGDMR	D	TFRPGGGDM	Cw*04:01	1.60	TFRPGGGDM	Cw*04:01	1.15	
		9	GMDGPKVKQWPLTEEKIK	A1	MDGPKVKQW	B*44:03	0.50	MDGPKVKQW	B*44:03	1.37	
		10	PLTSLKSLFGNDPLSQ	D	KSLFGNDPL	A*02:01	1.40	KSLFGNDPL	A*02:01	31.04	
			PLTSLKSLFGNDPLSQ	D	KSLFGNDPL	Cw*08:02	1.20				
			*FKGPRKIIKCFNCGKEGHI*	D							
		11	HERIEVKDTKEALEKI	D	EVKDTKEAL	B*14:02	1.40				
			HERIEVKDTKEALEKI	D	IEVKDTKEA	B*44:03	1.99	IEVKDTKEA	B*44:03	13.12	
E94	A*34:02	12	KIEEIQNKSKQKTQQAAA	A1	EIQNKSKQK	A*34:02	1.03	EIQNKSKQK	B*44:03	35.84	
	A*74:01	13	NHPSCVWLEAQEEEEVGF	A1	LEAQEEEEV	B*44:15	1.70	LEAQEEEEV	B*44:04	12.09	LEAQEEEEV
	B*44:03	14	HQDPIPKQPSSQPRGD	D	HQDPIPKQP	Cw*04:01	0.60	HQDPIPKQP	Cw*04:01	7.89	
	B*58:02							LEAQEEEEV	Cw*06:02	8.72	
	Cw*04:01										
	Cw*06:02										
E95	A*23:01	15	VAVHVASGYIEAEVIPA	A1	VAVHVASGY	Cw*16:01	1.50	VAVHVASGY	A*23:01	30.27	
	A*74:01	17	KRWIILGLNKIVRMYSPV	A1	WIILGLNKI	A*23:01	0.60	WIILGLNKI	A*23:01	6.99	
	B*44:03	16	KRWIILGLNKIVRMYSPV	A1	IILGLNKIV	A*74:01	0.60	IILGLNKIV	B*44:03	28.76	
	B*15:10	17	NMMLNIVGGHQAAMQMLK	A1	HQAAMQMLK	B*15:10	0.17				HQAAMQMLK
	Cw*04:01		NMMLNIVGGHQAAMQMLK	A1	HQAAMQMLK	A*74:01	0.90				
	Cw*16:01		NMMLNIVGGHQAAMQMLK	A1	HQAAMQMLK	Cw*04:01	1.30	HQAAMQMLK	Cw*04:01	26.63	
		18	KNWMTETLLVQNANPDCK	A1	TETLLVQNA	B*44:15	0.09	TETLLVQNA	B*44:03	5.85	
			KNWMTETLLVQNANPDCK	A1	KNWMTETLL	A*23:01	0.80	KNWMTETLL	A*23:01	25.75	
		19	FRDYVDRFFKTLRAEQA	A1	FRDYVDRFF	Cw*04:01	0.03	FRDYVDRFF	Cw*04:01	4.56	
			FRDYVDRFFKTLRAEQA	A1	FRDYVDRFF	A*23:01	0.60	FRDYVDRFF	A*23:01	4.56	
			FRDYVDRFFKTLRAEQA	A1	FRDYVDRFF	Cw*04:01	1.10	FRDYVDRFF	Cw*04:01	0.68	
		20	GATLEEMMTACQGVGGPGH	A1	EEMMTACQG	B*44:03	0.25	EEMMTACQG	B*44:03	0.97	
		21	LRALGPGATLEEMMTA	A1	RALGPGATL	B*15:10	1.80	RALGPGATL	B*44:01	0.56	
			LRALGPGATLEEMMTA	A1	RALGPGATL	Cw*04:01	0.60	RALGPGATL	Cw*04:01	0.56	
		22	FFKTLRAEQATQEVKNWM	A1	AEQATQEVK	B*44:03	0.15	AEQATQEVK	B*44:03	8.59	
		23	MEKEGKISKIGPENPY	A1	SKIGPENPY	B*15:03	0.50	SKIGPENPY	A*23:01	35.68	
			MEKEGKISKIGPENPY	A1	SKIGPENPY	B*15:10	0.50	SKIGPENPY	B*44:03	9.49	
		25	WVKVIEEKAFSPEVIPMF	A1	AFSPEVIPMF	A*23:01	0.40	AFSPEVIPM	A*23:01	5.95	
			WVKVIEEKAFSPEVIPMF	A1	WVKVIEEKA	A*23:01	1.70	WVKVIEEKA	A*23:01	17.53	
			WVKVIEEKAFSPEVIPMF	A1	EEKAFSPEV	B*44:03	0.80	EEKAFSPEV	B*44:03	3.38	
			WVKVIEEKAFSPEVIPMF	A1	EEKAFSPEV	B*44:15	0.03				EEKAFSPEV
			WVKVIEEKAFSPEVIPMF	A1	FSPEVIPMF	Cw*04:01	0.50	FSPEVIPMF	Cw*04:01	1.63	FSPEVIPMF
			WVKVIEEKAFSPEVIPMF	A1	FSPEVIPMF	Cw*16:01	0.50	FSPEVIPMF	A*23:01	0.46	
			WVKVIEEKAFSPEVIPMF	A1	KAFSPEVIP	Cw*16:01	1.20	KAFSPEVIP	B*4403	35.68	
		25	HQMKDCTERQANFLGKIW	A1	RQANFLGKI	B*4403	1.00	RQANFLGKI	B*4403	11.67	
		26	PMFSALSEGATPQDLNMM	A1	SEGATPQDL	B*44:03	0.80	SEGATPQDL	B*4403	0.33	
		27	HLARNCRAPRKKGCWK	A1	HLARNCRAP	A*74:01	0.60	HLARNCRAP	A*23:01	35.46	
			*LVQNANPDCKSILRAL*	A1							
		28	VATLYCVHQRIDVKDTK	A1	ATLYCVHQR	A*74:01	0.90	ATLYCVHQR	A*23:01	21.03	
		29	KIEEIQNKSKQKTQQAAA	A1	EIQNKSKQK	A*74:01	1.03	EIQNKSKQK	C*04:01	10.98	
		30	AGPIPPGQMREPRGSDIA	A1	AGPIPPGQM	B*15:10	0.60	AGPIPPGQM	C*04:01	2.02	
			*SKQKTQQAAADTGNSSKV*	A1							
E913	A*02:01	31	LWQRPLVTIKIGGQLKEA	D	LWQRPLVTI	A*02:01	1.60	LWQRPLVTI	A*02:01	11.25	
	A*34:02		LWQRPLVTIKIGGQLKEA	D	QRPLVTIKI	Cw*06:02	0.70	QRPLVTIKI	Cw*06:02	16.29	
	B*45:01		LWQRPLVTIKIGGQLKEA	D	WQRPLVTIK	B*47:01	1.90	WQRPLVTIK	B*45:01	19.93	
	B*47:01	32	TVPVKLKPGMDGPKVKQW	A1	LKPGMDGPK	A*34:02	0.90	LKPGMDGPK	Cw*06:02	26.76	
	Cw*06:02										
	Cw*16:01										
E914	A*01:01	33	DKWASLWNWFSITQWLWY	D	FSITQWLWY	A*01:01	0.06	FSITQWLWY	B*07:02	24.05	FSITQWLWY
	A*02:01		DKWASLWNWFSITQWLWY	D	KWASLWNWF	Cw*04:07	1.20				
	B*07:02		DKWASLWNWFSITQWLWY	D	SLWNWFSIT	A*02:01	1.66				
	B*44:15	34	PVDPDEVEKATEGENNSL	A1	ATEGENNSL	A*01:01	1.74				
	Cw*04:07										
	Cw*07:02										
L91	A*02:01	35	EQMHTDIISLWDQSLK	A1	IISLWDQSLK	A*03:01	1.90	IISLWDQSL	A*03:01	20.55	ISLWDQSLK
	A*03:01		EQMHTDIISLWDQSLK	A1	MHTDIISLW	B*58:02	0.90	MHTDIISLW	A*03:01	23.95	
	B*53:01		EQMHTDIISLWDQSLK	A1	MHTDIISLW	Cw*06:02	1.30	MHTDIISLW	Cw*06:02	3.72	
	B*58:02		EQMHTDIISLWDQSLK	A1	QMHTDIISL	A*02:01	1.90				
	Cw*04:01		EQMHTDIISLWDQSLK	A1	QMHTDIISL	B*5301	1.20	QMHTDIISL	B*5301	18.52	
	Cw*06:02	36	LETSEGCKQIIGQLQPAI	D	ILAQLQPAI	A*02:01	0.40				
		37	SGGKLDAWEKIRLRPGGK	A1	KIRLRPGGK	A*03:01	0.25	KIRLRPGGK	A*03:01	0.06	KIRLRPGGK
		38	LETTEGCQQIMEQLQPAL	A1	IMEQLQPAL	A*03:01	0.40	IMEQLQPAL	A*03:01	21.06	
			LETTEGCQQIMEQLQPAL	A1	IMEQLQPAL	Cw*04:01	0.80	IMEQLQPAL	Cw*04:01	0.19	
			LETTEGCQQIMEQLQPAL	A1	QIMEQLQPA	A*02:01	0.70				
		39	ERILSTCLGRSAEPVPL	A1	RSAEPVPL	B*58:02	0.12				
			ERILSTCLGRSAEPVPL	A1	RILSTCLGR	A*03:01	0.90	RILSTCLGR	A*03:01	0.11	
			ERILSTCLGRSAEPVPL	A1	CLGRSAEPV	A*02:01	2.00				
		40	LVGPTPVNIIGRNMLTQI	A1	LVGPTPVNI	A*02:01	1.61	LVGPTPVNI	:01	4.69	
		41	CKQIIGQLQPAIQTGSEEL	D	QIIGQLQPA	A*02:01	1.80				
			CKQIIGQLQPAIQTGSEEL	D	AIQTGSEEL	A*03:01	1.50	AIQTGSEEL	A*03:01	21.04	
			CKQIIGQLQPAIQTGSEEL	D	IIGQLQPAI	A*03:01	1.10				
		42	PAIQTGSEELRSLYNTVA	D	AIQTGSEEL	A*03:01	1.50	AIQTGSEEL	Cw*06:02	3.74	
			PAIQTGSEELRSLYNTVA	D	LRSLYNTVA	Cw*06:02	0.70	LRSLYNTVA	Cw*06:02	5.48	
L92	A*02:01	43	NDIQKLVGKLNWASQIYP	D	KLNWASQIY	A*30:02	0.50	KLNWASQIY	A*30:02	5.48	KLNWASQIY
	A*30:02		NDIQKLVGKLNWASQIYP	D	KLVGKLNWA	A*02:01	0.60	KLVGKLNWA	A*02:01	1.22	
	B*44:03		NDIQKLVGKLNWASQIYP	D	KLNWASQIY	A*02:01	0.90	KLNWASQIY	A*02:01	11.10	
	B*14:02		NDIQKLVGKLNWASQIYP	D	KLNWASQIY	Cw*04:01	1.80	KLNWASQIY	Cw*04:01	13.24	
	Cw*04:01	44	LVVKTYWGLHTGEREWHL	D	LVVKTYWGL	A*02:01	1.70	LVVKTYWGL	A*02:01	0.95	
	Cw*08:02		LVVKTYWGLHTGEREWHL	D	VVKTYWGLH	A*30:02	1.50				
		45	SLVNRVRQGYSPLSFQTL	D	NRVRQGYSPL	B*14:02	0.12				
			SLVNRVRQGYSPLSFQTL	D	YSPLSFQTL	Cw*04:01	0.70	YSPLSFQTL	Cw*04:01	2.47	
			SLVNRVRQGYSPLSFQTL	D	RQGYSPLSF	A*30:02	1.20	RQGYSPLSF	A*30:02	4.51	RQGYSPLSF
			SLVNRVRQGYSPLSFQTL	D	RQGYSPLSF	Cw*04:01	1.40	RQGYSPLSF	Cw*04:01	3.12	
		46	TLPCRIKQIINMWQGV	D	CRIKQIINM	A*02:02	0.40	CRIKQIINM	A*30:02	28.36	CRIKQIINM
		47	MRVRGIQRNYQHLWRW	D	RNYQHLWRW	B*44:03	0.40	RNYQHLWRW	B*44:03	3.17	
		48	GEMKNCSFNITTEIRDKK	D	EMKNCSFNI	B*44:03	0.30	EMKNCSFNI	B*44:03	32.11	
		49	NVTENFNMWKNNMVEQMH	D	NFNMWKNNM	Cw*04:01	1.06	NFNMWKNNM	Cw*04:02	4.53	
			NVTENFNMWKNNMVEQMH	D	TENFNMWKNNM	B*44:03	1.81	TENFNMWKN	B*44:03	7.71	
		50	WLIDRIRERAEDSGNESE	D	WLIDRIRER	A*02:01	2.00	WLIDRIRER	A*02:01	4.68	
L94	A*3402	51	LIHLHYFDCFSDSAIRKA	A1	YFDCFSDSA	Cw*04:01	0.90	YFDCFSDSA	Cw*04:01	2.52	
	A*7401		LIHLHYFDCFSDSAIRKA	A1	YFDCFSDSA	Cw*06:02	1.60	YFDCFSDSA	Cw*06:02	14.91	
	B*4403		LIHLHYFDCFSDSAIRKA	A1	HLHYFDCFSDSAIR	A*7401	1.40				
	B*5802		LIHLHYFDCFSDSAIRKA	A1	FSDSAIRKA	Cw*04:01	1.10	FSDSAIRKA	Cw*04:01	0.78	
	Cw*0401	52	HLARNCRAPRKKGCWK	A1	ARNCRAPRK	A*3402	1.10				
	Cw*0602		HLARNCRAPRKKGCWK	A1	HLARNCRAP	A*3402	1.50				
			HLARNCRAPRKKGCWK	A1	HLARNCRAP	A*74:01	0.60				
		53	SKQKTQQAAADTGNSSKV	A1	AADTGNSSK	A*3402	1.15				
		54	HQDPIPKQPSSQPRGD	D	HQDPIPKQP	Cw*04:01	0.60	HQDPIPKQP	Cw*04:01	7.89	
L95	A*23:01	55	KRWIILGLNKIVRMYSPV	A1	WIILGLNKI	A*23:01	0.60	WIILGLNKI	A*23:01	6.99	
	A*74:01		KRWIILGLNKIVRMYSPV	A1	IILGLNKIV	A*74:01	0.60	IILGLNKIV	Cw*04:01	2.81	
	B*44:03	56	NMMLNIVGGHQAAMQMLK	A1	GHQAAMQML	B*15:10	0.40				
	B*15:10		NMMLNIVGGHQAAMQMLK	A1	HQAAMQMLK	A*74:01	0.90				HQAAMQMLK
	Cw*04:01		NMMLNIVGGHQAAMQMLK	A1	HQAAMQMLK	Cw*04:01	1.30	HQAAMQMLK	Cw*04:01	26.63	
	Cw*16:01	57	EVNIVTDSQYALGIIQA	A1	EVNIVTDSQ	B*44:03	0.50	EVNIVTDSQ	B*44:03	19.78	
		58	AYETEMHNVWATHACV	A1	TEMHNVWAT	B*44:03	0.40	TEMHNVWAT	B*44:03	0.88	YETEMHNVW
			AYETEMHNVWATHACV	A1	MHNVWATHA	B*15:10	0.90	MHNVWATHA	Cw*04:01	11.32	
			AYETEMHNVWATHACV	A1	MHNVWATHA	B*44:03	0.90	MHNVWATHA	B*44:04	22.06	
		59	AAEWDRLHPVHAGPI	A1	AAEWDRLHP	B*44:03	0.50	AAEWDRLHP	B*44:03	34.74	AEWDRLHPV
			AAEWDRLHPVHAGPI	A1	LHPVHAGPI	B*15:10	0.15	AAEWDRLHP	A*23:01	34.79	
			AAEWDRLHPVHAGPI	A1	RLHPVHAGP	A*74:01	1.50	AAEWDRLHP	Cw*04:01	6.59	
		60	LRALGPGATLEEMMTA	A1	RALGPGATL	B*15:10	0.60	RALGPGATL	A*23:01	8.98	
			LRALGPGATLEEMMTA	A1	RALGPGATL	Cw*04:01	0.60	RALGPGATL	Cw*04:01	0.56	
		61	FFKTLRAEQATQEVKNWM	A1	AEQATQEVK	B*44:03	0.15	AEQATQEVK	B*44:03	8.59	
		62	GTTSTPQEQIGWMTGNPPI	A1	QEQIGWMTG	B*44:15	0.65	QEQIGWMTG	B*44:03	1.85	
			GTTSTPQEQIGWMTGNPPI	A1	GWMTGNPPI	A*23:01	1.40	GWMTGNPPI	A*23:01	0.75	
		63	WVKVIEEKAFSPEVIPMF	A1	EEKAFSPEV	B*44:15	0.62				
			WVKVIEEKAFSPEVIPMF	A1	EEKAFSPEV	B*44:03	0.80	EEKAFSPEV	B*44:03	3.38	EEKAFSPEV
			WVKVIEEKAFSPEVIPMF	A1	WVKVIEEKA	A*23:01	1.70	WVKVIEEKA	A*23:01	34.76	
			WVKVIEEKAFSPEVIPMF	A1	FSPEVIPMF	Cw*04:01	0.50	FSPEVIPMF	Cw*04:01	1.63	
			WVKVIEEKAFSPEVIPMF	A1	AFSPEVIPM	Cw*16:01	0.70	AFSPEVIPM	Cw*04:01	0.28	
			WVKVIEEKAFSPEVIPMF	A1	KAFSPEVIP	Cw*16:01	1.20	KAFSPEVIP	Cw*04:01	7.47	
		64	TVYYGVPVWKDAETTLF	A1	TVYYGVPVW	A*74:01	0.17	TVYYGVPVW	B*44:03	7.96	VYYGVPVWK
			TVYYGVPVWKDAETTLF	A1	TVYYGVPVW	Cw*16:01	1.10	TVYYGVPVW	Cw*04:01	1.25	
			TVYYGVPVWKDAETTLF	A1	WKDAETTLF	B*15:10	0.90				
			TVYYGVPVWKDAETTLF	A1	VWKDAETTL	A*23:01	0.60	WKDAETTLF	A*23:01	4.36	
			TVYYGVPVWKDAETTLF	A1	VWKDAETTL	Cw*04:01	0.60	VWKDAETTL	Cw*04:01	1.97	
		65	LRWGTMILGMIIICSAA	A1	RWGTMILGM	A*23:01	0.90	RWGTMILGM	A*23:01	3.97	
			LRWGTMILGMIIICSAA	A1	RWGTMILGM	Cw*04:01	0.94	RWGTMILGM	Cw*04:01	1.34	
		66	GHQAAMQMLKDTINEEAA	A1	HQAAMQMLK	A*74:01	0.90	HQAAMQMLK	A*23:01	32.48	
		67	IKQGPKEPFRDYVDRFFK	A1	FRDYVDRFF	A*23:01	0.60	FRDYVDRFF	A*23:01	10.14	
			IKQGPKEPFRDYVDRFFK	A1	FRDYVDRFF	Cw*04:01	0.60	FRDYVDRFF	Cw*04:01	0.68	
		68	FRDYVDRFFKTLRAEQA	A1	FRDYVDRFF	A*23:01	0.60	FRDYVDRFF	A*23:01	10.14	
			*LVQNANPDCKSILRAL*	A1							
		69	MREPRGSDIAGTTSTPQEQI	A1	MREPRGSDI	B*15:10	2.00	MREPRGSDI	Cw*04:01	1.95	
		70	EKIRLRPGGKKKYRLKHL	A1	RLRPGGKKK	A*74:01	0.28	RLRPGGKKK	A*23:01	28.99	
		71	VATLYCVHQRIDVKDTK	A1	ATLYCVHQR	A*74:01	0.90	ATLYCVHQR	B*44:03	28.12	
		72	LFCASDAKAYETEMHNVW	A1	SDAKAYETEMHNVW	B*44:03	0.31	KAYETEMHN	B*44:03	35.92	
L913	A*02:01	73	PPLVKLWYQLEKEPIIGA	D	LVKLWYQLE	A*34:01	0.50	LVKLWYQLE	A*02:01	18.93	
	A*34:02		PPLVKLWYQLEKEPIIGA	D	KLWYQLEKEPIIGA	A*02:01	1.47	WYQLEKEPI	A*02:01	18.24	
	B*45:01		PPLVKLWYQLEKEPIIGA	D	QLEKEPIIG	B*45:01	0.90	QLEKEPIIG	B*45:01	17.25	
	B*47:01		PPLVKLWYQLEKEPIIGA	D	YQLEKEPII	A*02:01	0.80	YQLEKEPII	A*02:01	0.25	
	Cw*06:02		PPLVKLWYQLEKEPIIGA	D	YQLEKEPII	B*47:01	1.20	YQLEKEPII	Cw*06:02	2.05	
	Cw*16:01	74	KWKPKMIGGIGGFIKVR	D	MIGGIGGFIK	A*34:02	0.20				
			KWKPKMIGGIGGFIKVR	D	KMIGGIGGF	A*02:01	1.00	KMIGGIGGF	A*02:01	1.55	
			KWKPKMIGGIGGFIKVR	D	KMIGGIGGF	B*47:01	1.10	KMIGGIGGF	Cw*06:02	5.37	
		75	VIWGKTPKFRLPIQKETW	D	IVIWGKTPK	A*34:02	0.15				
			VIWGKTPKFRLPIQKETW	D	KTPKFRLPI	Cw*16:01	1.10	KTPKFRLPI	A*02:01	4.43	
			VIWGKTPKFRLPIQKETW	D	VIWGKTPKF	A*34:02	1.00	VIWGKTPKF	Cw*06:02	7.82	
		76	RQANFLGKIWPSHKGR	D	RQANFLGKI	B*47:01	0.40				
			RQANFLGKIWPSHKGR	D	RQANFLGKI	Cw*06:02	2.00	RQANFLGKI	Cw*06:02	9.23	
			RQANFLGKIWPSHKGR	D	FLGKIWPSH	A*34:02	1.10	RQANFLGKI	B*45:01	7.03	
		77	KIEELREHLLRWGFTTPDK	D	REHLLRWGF	B*47:01	0.03	REHLLRWGF	A*02:01	20.74	
			KIEELREHLLRWGFTTPDK	D	REHLLRWGF	B*45:01	0.70	REHLLRWGF	B*45:01	1.11	
			KIEELREHLLRWGFTTPDK	D	LREHLLRWG	Cw*06:02	1.40	LREHLLRWG	Cw*06:02	19.44	
			KIEELREHLLRWGFTTPDK	D	HLLRWGFTT	A*02:01	1.20	HLLRWGFTT	A*02:01	0.20	
		78	GFAILKCKDKEFNGTGPCK	A1	KEFNGTGPC	B*45:01	1.50	KEFNGTGPC	B*45:01	1.25	
		79	AILNIPTRIRQGLERALL	D	IRQGLERAL	Cw*06:02	0.60	IRQGLERAL	Cw*06:02	0.71	
			AILNIPTRIRQGLERALL	D	AILNIPTRI	A*02:01	0.60				
			AILNIPTRIRQGLERALL	D	RQGLERALL	B*47:01	1.70	RQGLERALL	B*45:01	13.50	
		80	QKTELQAINLALQDSGLEV	D	LALQDSGLE	A*02:01	1.50	LALQDSGLE	A*02:01	22.65	
			QKTELQAINLALQDSGLEV	D	TELQAINLA	B*47:01	0.60	TELQAINLA	B*45:01	0.15	
			QKTELQAINLALQDSGLEV	D	QKTELQAIN	B*45:01	1.00	QKTELQAIN	B*45:01	12.04	
			QKTELQAINLALQDSGLEV	D	NLALQDSGL	A*34:02	1.50	NLALQDSGL	A*02:01	4.72	
		81	IIGRNLLTQIGCTLNFPI	D	IGCTLNFPI	A*02:01	0.90	IGCTLNFPI	A*02:01	4.79	
			IIGRNLLTQIGCTLNFPI	D	NLLTQIGCTLNFPI	A*02:01	1.63				
			IIGRNLLTQIGCTLNFPI	D	LLTQIGCTL	A*02:01	1.90	LLTQIGCTL	A*02:01	0.53	
			IIGRNLLTQIGCTLNFPI	D	TQIGCTLNF	Cw*16:01	1.40	TQIGCTLNF	Cw*06:02	2.26	
			IIGRNLLTQIGCTLNFPI	D	LLTQIGCTL	Cw*16:01	0.90				
		82	KWKPKMIGGIGGFIKVR	D	KMIGGIGGF	A*02:01	1.00	KMIGGIGGF	A*02:01	1.55	
		83	LWQRPLVTIKIGGQLKEA	D	LWQRPLVTI	A*02:01	1.60	LWQRPLVTI	A*02:01	11.25	
			LWQRPLVTIKIGGQLKEA	D	QRPLVTIKI	Cw*06:02	0.70	LWQRPLVTI	Cw*06:02	0.74	
		84	LKEALLDTGADDTVLEEI	D	LKEALLDTG	B*45:01	1.20	LKEALLDTG	B*45:01	12.15	
		85	KRQEILDLWVYHTQGYF	A1	QEILDLWVY	B*45:01	1.70	QEILDLWVY	B*45:01	1.02	
			KRQEILDLWVYHTQGYF	A1	QEILDLWVY	B*47:01	0.90				
			KRQEILDLWVYHTQGYF	A1	RQEILDLWV	Cw*06:02	1.10	RQEILDLWV	Cw*06:02	21.81	
			KRQEILDLWVYHTQGYF	A1	ILDLWVYHT	A*02:01	0.70	ILDLWVYHT	B*45:01	13.80	
			*IYSLIEESQNQQEKNEQEL*	D							
L914	A*01:01	86	SFNCGGEFFYCNTSGLF	A1	SFNCGGEFFY	A*01:01	0.25				
	A*02:01		SFNCGGEFFYCNTSGLF	A1	SFNCGGEFF	Cw*04:07	1.30	SFNCGGEFF	B*44:03	7.72	
	B*07:02		SFNCGGEFFYCNTSGLF	A1	SFNCGGEFF	Cw*07:02	1.10	SFNCGGEFF	B*07:02	22.45	
	B*44:03		SFNCGGEFFYCNTSGLF	A1	GEFFYCNTS	B*44:03	1.30	GEFFYCNTS	B*44:03	1.80	
	Cw*04:07	87	MEKEGKISKIGPENPY	A1	KEGKISKIGPENPY	B*44:03	1.30	KISKIGPEN	B*44:03	39.08	
	Cw*07:02		MEKEGKISKIGPENPY	A1	ISKIGPENP	A*01:01	1.80	ISKIGPENP	B*07:02	27.27	
		88	ARKNRRRRWRARQRQI	A1	RRWRARQRQ	Cw*07:02	0.60	RRWRARQRQ	Cw*07:02	19.17	

Experimentally mapped peptides for all participants and their cognate computational core 9-mer and a single 14-mer epitope sequence with scores. Peptides shown in italic text were not algorithmically predicted as binders. Multiple computational predictions contained in a single experimental peptide were counted as a single hit. Participant’s identifiers (ID) beginning with E or L represent early or late time sampling points respectively

**Table 3:** Peptides not predicted

Participant’s Identification	Participant’s HLA Alleles	Experimental Peptide Sequence
E92	A*02:01	FKGPRKIIKCFNCGKEGHI
	A*30:02	
	B*44:03	
	B*14:02	
	Cw*04:01	
	Cw*08:02	
E95	A*23:01	LVQNANPDCKSILRAL (both time points)
	A*74:01	SKQKTQQAAADTGNSSKV
	B*44:03	
	B*15:10	
	Cw*04:01	
	Cw*16:01	
L913	A*02:01	IYSLIEESQNQQEKNEQEL
	A*34:02	
	B*45:01	
	B*47:01	
	Cw*06:02	
	Cw*16:01	

Experimentally mapped peptides that were not predicted by NetMHCpan4.0 as binders. Participant’s identifiers beginning with E or L represent early or late time sampling points respectively

**Fig. 3:**
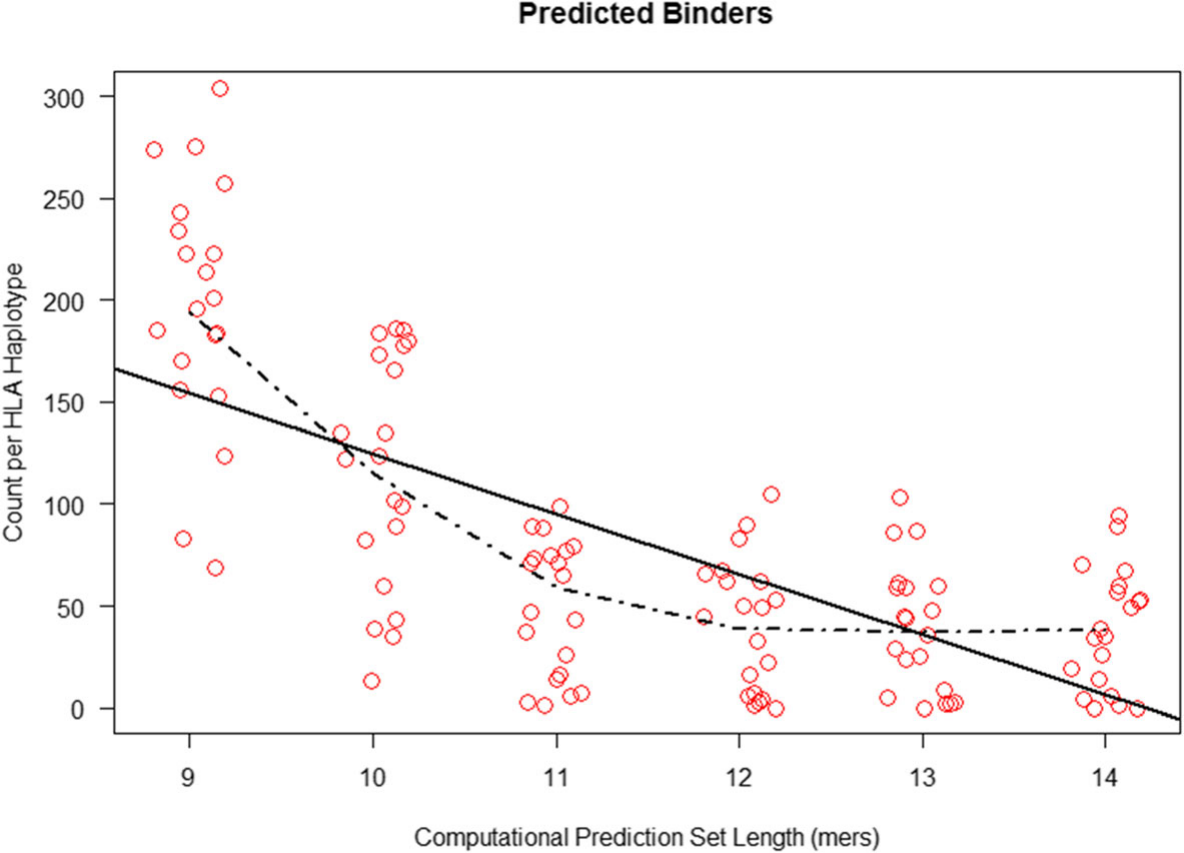
Computational epitope prediction**.** NetMHCpan4.0 set length plotted against the number of predicted binders per HLA type shows that the number of predictions reduces as the input set length increases. The dotted line is the trend line, whereas the solid line is the line of best fit. The core 9mer epitope sequence was similar across 9mer through 14mer set length except for one 14-mer peptide (hit 72 in Table 2)

Comparison of the various epitope prediction length set showed that the 9mer setting was ideal for NetMHCpan4.0. The number of predictions were 88, 79, 55, 39, 39 and 37 hits out of 93 for 9, 10, 11, 12, 13 and 14-mer epitopes respectively. Increasing the prediction length from 9mer through 14mer resulted in a smaller number of predicted binders as illustrated in Fig. 3. Since we held the assumption that our wet experimental data was the gold standard we evaluated the sensitivity and specificity of NetMHCpan4.0.The computational predictor had more predicted binders than those determined by the experimental mapping as presented in the confusion matrix in Table 4. The experimental positive’s count also shown in Table 2 under column “Hit No” shows the test peptide count (1through 88) that contained the computational 9-mer sequence. Multiple computational epitopes may be contained in a single experimental peptide, as shown in the column “NetMHCpan4.0 9-mer Epitope Prediction” in Table 2. Overall HIV-1 Clade A 9-mer predictions were fewer in number than clade D (Fig. 2, c) though the difference did not approach statistical significance.

**Table 4:** Experimental and computational 9mer peptide confusion matrix

	Experimental Positive	Experimental Negative
**Computational Positive** (≥1 epitope(s) contained in a single experimental peptide sequence)	**True Positive (88)** (Hits in table 2)	**False Positive (37)**
**Computational Negative**	**False Negative (5)**	**True Negative (627)**

The total number of peptides experimentally tested were 757 and these are broken down to show the fractions from both the experimental testing and NetMHCpan4.0 computational predictions

### Comparison of experimentally mapped epitopes with *in-silico* prediction

**Fig. 4:**
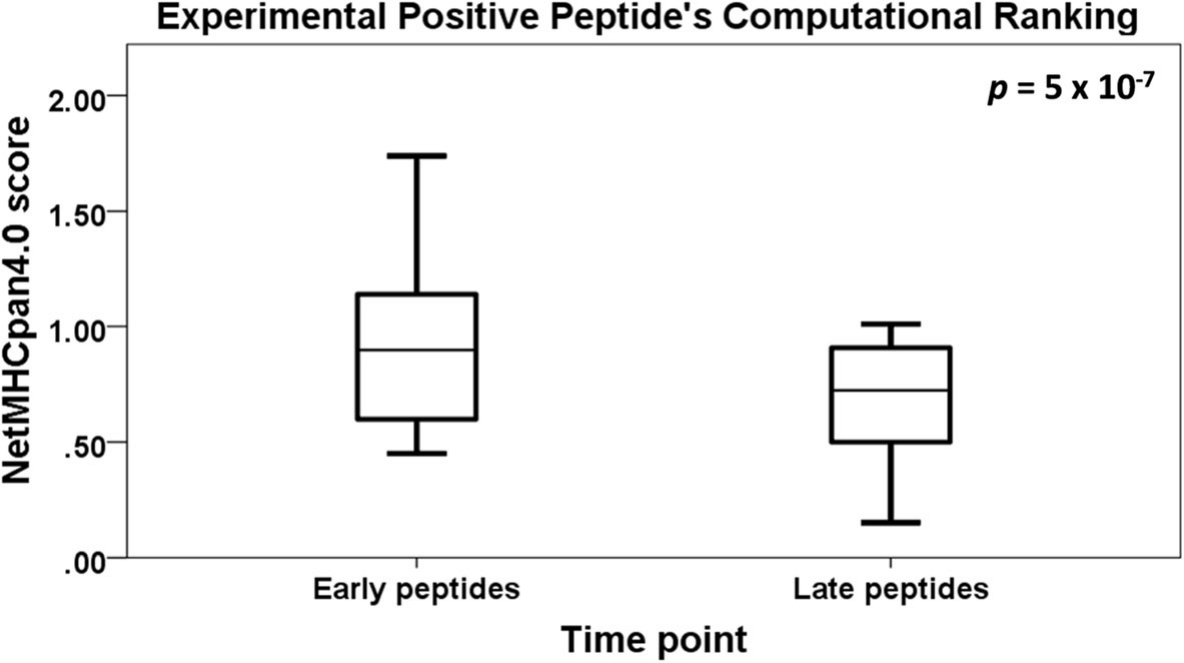
Early versus Late Peptides**.** Experimentally mapped peptides at baseline (*n* = 34) and at least 12 months later (*n* = 34) were compared using the 9-mer computational NetMHCpan4.0 scores of the hits. The lower the computational score the stronger the predicted binding. Late peptides were significantly stronger binders than early peptides (Wilcoxon signed rank test, *p* = 0.0000005)

**Fig. 5:**
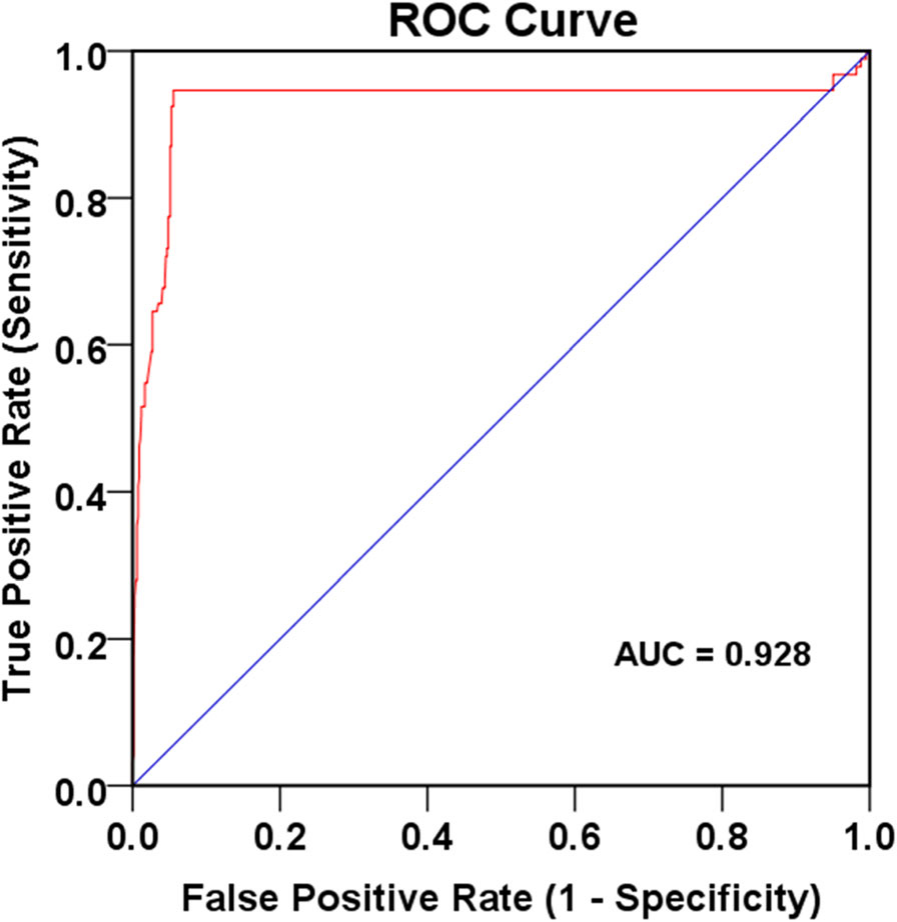
ROC plot**.** False versus true positive rate for all 9-mer and a single 14-mer test peptides across the 22 test HLA class I types. The diagonal line shows the random guess whereas the red curve shows the observed experimentally mapped epitopes versus the NetMHCpan4.0 expected predictions

The experimental peptide mapping data was derived from a baseline time point corresponding to HIV-1 Fiebig stages IV, V and VI (Table 1) and a later time point. Ninety-three (*n* = 93) epitopes were experimentally mapped of which 12 were recognized at both baseline and later time points, 34 only at baseline and 54 only at the later time point. Comparison of the ranked computational score for Netmhcpan4.0 binders of early (*n* = 34) versus later peptides showed that the later time point predictions were stronger binders reaching statistical significance (Wilcoxon signed rank *p*-value = 0.0000005) (Fig. 4). NetMHCpan4.0 ranked binders as those predicted to be in the top 2% and assigned a score of 0.2 or below. Any binder within the top 0.5% and assigned a score of 0.05 or below was ranked as a strong binder. Considering only the 9-mer computational predictions, peptides that were derived from the same 17-mer experimental peptide were determined by a BLAST mapping to their derivative sequences. The 17-mer peptides were then classified into a confusion matrix (Table 4) as true positives, false positives, true negatives or false negatives. From the classification the true positive rate (sensitivity) was plotted against the false positive rate (1-specificity) using an ROC curve and the AUC attained reached 0.928 (Fig. 5). Only 9-mer length epitopes were considered in the ROC analysis as increasing the length to 10-mer through 14mer NetMHCpan4.0 predictions neither raised the number of predicted binders nor improved the hit rate as all their predictions contained the sequence already predicted in the 9-mer set except 1, 14-mer peptide (hit 72 in Table 2). Comparison of the ELISPOT magnitude of response (spot forming units) did not show any association to either NetMHCpan4.0 scores or MHCflurry1.2.0 affinity values. Similarly a comparison of the latter 2 computational predictors did not show any association between their assigned “affinity” values. NetMHCpan4.0 registered the highest concordance to the wet experiments followed by MHCflurry1.2.0.

## Discussion

In this analysis we showed that the computational method NetMHCpan4.0 predicted 95% of previously experimentally mapped HIV-1 epitopes in 6 HIV-1 infected individuals expressing a total of 22 different HLA class I alleles. In our IFN-γ ELISPOT assays we evaluated 757 17mer peptides overlapping by 11 amino acids and covering the whole HIV-1 subtype A1 and D consensus proteomes. Out of the 5 experimentally determined epitopes missed by the algorithm (Table 3), 4 were actually computationally predicted as binders but were not included for lack of concordance with the participant’s HLA alleles. About one third (37) of 125 total positive predictions were not experimentally supported in our tests. These do not necessarily represent false positives, as ELISPOT detection depends on the frequency of specific T cells in the participant’s repertoire, and we observed changes in dominant T cell specificities within a given participant between early and later time points after HIV-1 infection. A formal ROC evaluation of the score generated by NetMHCpan4.0 as a classifier for peptides recognised/not recognised by PBMC in IFN-g ELISPOT assays, produced an AUC of 0.928. Thus experimental confirmatory tests cannot be dropped altogether, however the NetMHCpan4.0 algorithm could provide a considerable saving of time and resources in verifying just the predicted epitopes.

As the participants had been enrolled in the acute/early phase of HIV-1 infection and we had observed intra-participant changes in epitope recognition between early and late time points after infection, we compared the binding scores of confirmed epitopes at these time points and found a statistically significant change towards recognition of higher binding peptides as the infection entered the chronic phase. This might represent better support of the T-cell response directed at more stable HLA/peptide complexes as the infection progresses into chronicity.

The NetMHCpan4.0 algorithm, which is based on binding affinity and integrates data on eluted naturally processed ligands, reflected optimal HLA class I binding for 9-mers, producing a decreasing number of predictions when the peptide size was increased from 9 to 11 amino acids. With a single exception, predicted binders between 11 and 14 amino acids included at least one 9mer predicted to bind on its own, suggesting a destabilizing effect of the extra amino acids beyond the canonical HLA class I binding pockets at positions 2 and 9 could account for fewer predictions.

Important limitations are the lack of predictions of HLA class II restricted epitopes, which might have contributed to a fraction of IFN-γ ELISPOT responses. Approximately 5% of the computational predictions may be false positives that only increase the size of planned wet experiments and approximately 1% of true positives may also be missed.

## Conclusion

In this analysis, using NetMHCpan4.0, MHCflurry and NetCTL to predict previously experimentally mapped epitopes, we demonstrate that the computational methods reliably predict an acceptable portion of binder epitopes. We recommend the use of such computational methods to reduce the size of experiments required cost associated.

## Data Availability

Most of the relevant data to support the manuscript has been included in the write-up. If any addition data is required will be availed once requested.

## Abbreviations

AUC: Area under the curve
CTL: Cytotoxic T lymphocytes
HIV-1: Human immunodeficiency virus type 1
HLA: Human leucocyte antigen
ROC: Receiver operator characteristic
